# An unanticipated cause of worsening dyspnoea: mitral stenosis induced by cardiac myxoma

**DOI:** 10.1093/ehjimp/qyaf024

**Published:** 2025-02-26

**Authors:** Deniz Mutlu, Hakan Yalman, Mehmet Semih Belpınar, Anıl Şahin, Zafer Akman, Davide Margonato

**Affiliations:** Minneapolis Heart Institute Foundation, Abbott Northwestern Hospital, 920 E 28th Street #100, Minneapolis, MN 55407, USA; Cerrahpasa Faculty of Medicine, Istanbul University-Cerrahpasa, Istanbul, 34098, Turkey; Cerrahpasa Faculty of Medicine, Istanbul University-Cerrahpasa, Istanbul, 34098, Turkey; Cerrahpasa Faculty of Medicine, Istanbul University-Cerrahpasa, Istanbul, 34098, Turkey; Department of Cardiology, Cumhuriyet University, Sivas, 58140, Turkey; Department of Internal Medicine, Yale University, New Haven, CT 06520, USA; Minneapolis Heart Institute Foundation, Abbott Northwestern Hospital, 920 E 28th Street #100, Minneapolis, MN 55407, USA

**Keywords:** myxoma, mitral stenosis, multi-modality imaging, cardiac MRI, cardiac tumour

A 57-year-old female with hypertension presented with worsening dyspnoea, over the last 3 months. Physical examination was notable for crackles on the bilateral lower lung zones and pretibial oedema. Echocardiography demonstrated normal left ventricular function and a hyperechoic polypoid mass measuring 35 × 76 mm attached to the inter-atrial septum (IAS), prolapsing into the left ventricle during diastole and resulting in severe mitral stenosis (*Figure 1A–D*) and severe pulmonary hypertension (systolic pulmonary arterial pressure: 90 mmHg). Magnetic resonance imaging (MRI) confirmed the presence of a large mass with high signal intensity and heterogenous components attached at the level of fossa ovalis with a thin pedicle, obstructing mitral valve opening; these characteristics were highly suspicious for a diagnosis of cardiac myxoma (*Figure 1E* and *F*). The mass was surgically removed by clean dissection along the IAS, and histological examination was consistent with atrial myxoma. Post-operatively, the patient recovered well. On follow-up echocardiography 1-month after the procedure, no residual mass was detected in the left atrium, and patient’s symptoms markedly diminished at 9-month follow-up.

**Figure 1:**
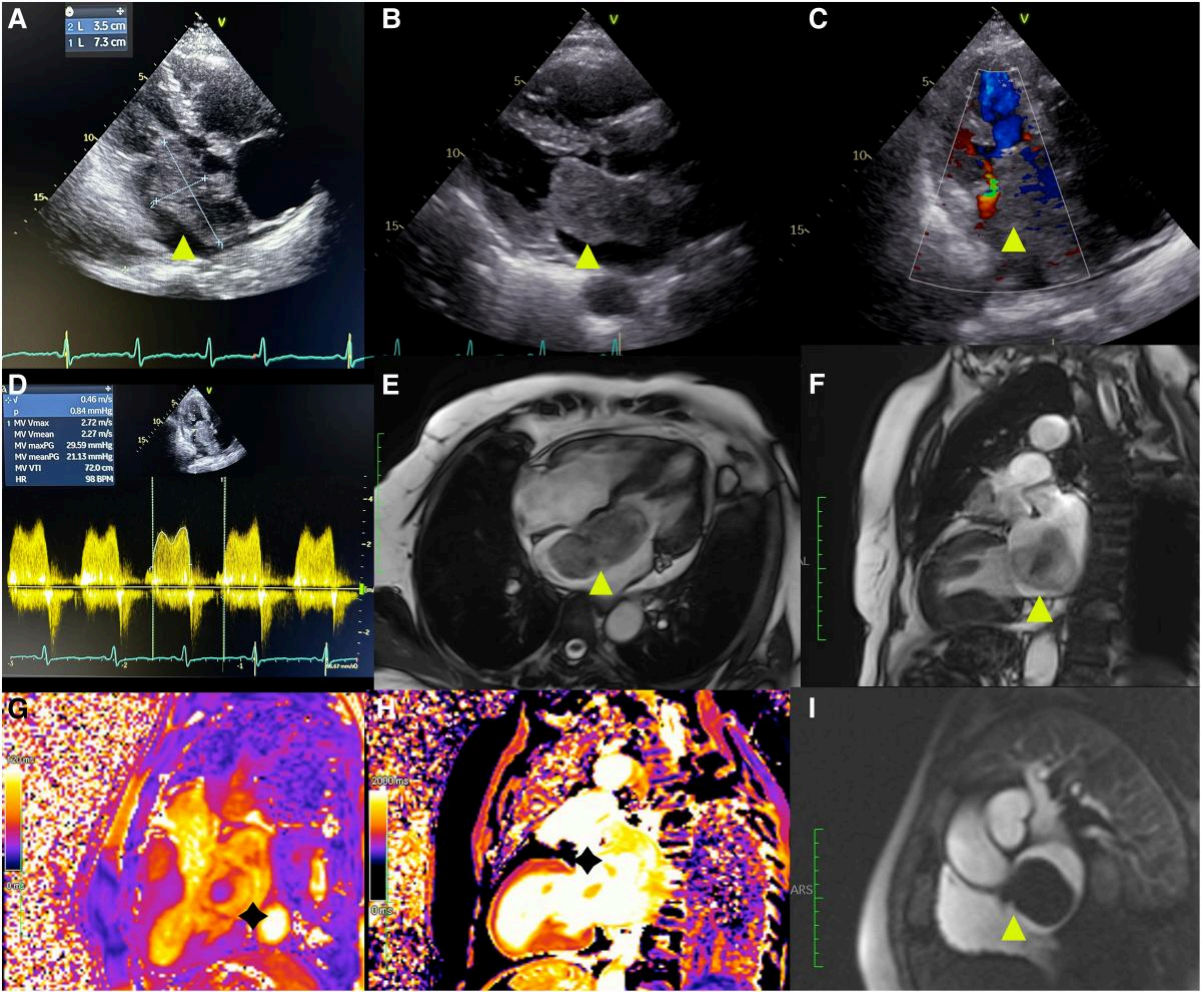
Echocardiography demonstrates a hyperechoic polypoid mass (arrowhead) obstructing the mitral valve in the (*A*) apical four-chamber, (*B*) parasternal long-axis, and (*C*) apical three-chamber views. (*D*) MV CW Doppler imaging shows an increased gradient (maximum MVG: 29 mmHg, mean MVG: 21 mmHg) due to the mass obstruction. Cardiac MRI demonstrates a mass with high signal intensity and heterogenous components on the horizontal long-axis gradient echo sequence: (*E*) four-chamber and (*F*) two-chamber views. In the T1 and T2 mapping sequences, a cystic mass (asterisk) was demonstrated with increased (*G*) T2 time and (*H*) native T1 value. (*I*) Turbo Flash sequence demonstrated the mass effect of the cystic lesion causing mitral valve obstruction. CW, continuous wave; MV, mitral valve; MVG, MV gradient; VTI, velocity time integral.

Cardiac myxomas are infrequent, with an incidence of 0.5 per million individuals per year. However, they can result in life-threatening complications such as obstructive symptoms, embolic phenomenon, and constitutional symptoms. Left atrium is the most common location, and it should be differentiated from a thrombus and other masses. MRI has an exceptional tissue characterization capability to determine mass origin and local invasion. Surgical excision is the preferred treatment modality, particularly in cases of symptomatic valve obstruction.

## Data Availability

No new data were generated or analysed in support of this research.

## Notes

**Consent:** We confirm that we have written consent for submission and publication of image and associated text.

**Funding**: None declared.

## Lead author biography

Deniz Mutlu, MD, serves as a senior research scholar at the Minneapolis Heart Institute Foundation, Center for Coronary Artery Disease, Minneapolis, MN, USA, under the guidance of Dr Emmanouil Brilakis. Graduating as valedictorian, Dr Mutlu earned his MD degree from Acibadem University, followed by the completion of his cardiology fellowship at Istanbul University-Cerrahpasa in Istanbul, Turkey. His primary research focus lies in investigating the clinical outcomes associated with complex percutaneous coronary interventions, notably bifurcation stenting and chronic total occlusion interventions. He is passionate about imaging applications in cardiology practice, including intravascular imaging, cardiac CTA, and MRI.

